# 

**Published:** 1823-04

**Authors:** 


### MONTHLY CATALOGUE OF MEDICAL BOOKS.

*** *It having been hinted to us that gentlemen, sending copies of their Works, prefer having their titles given at length, it is our intention, hereafter, to comply with this suggestion: but it is to be observed, that no books can be entered on this list except those sent to us for the purpose;—in the lists hitherto transmitted, we find that the names of works have frequently been given as published, which have not appeared for weeks, or even months after.*

History of the Method of Cure in the various Species of Epilepsy; being the second Part of the second Volume of a Treatise on Nervous Diseases. By JOHN COOKE, M.D. F.R.S. F.A.S. Fellow of the Royal College of Physicians, and late Physician to the London Hospital.—pp. 235. London, 1823.

Remarks on the Yellow Fever of the South and East Coasts of Spain; comprehending Observations made on the Spot, by actual Survey of Localities, and rigorous Examination of Fact at original Sources of Information. By THOMAS O'HALLORAN, M.D. Member of the Medical Academies of Madrid and Barcelona. —pp. 208. London, 1823.

An Address, delivered in the Guildhall at Plymouth, on the 6th Day of December, 1821, at the first General Meeting of the Subscribers to the Plymouth Eye Dispensary. Published at the Request of the Committee, (with an Appendix, and List of Subscribers,) for the Benefit of the said Institution. By JOHN BUTTER, M.D. F.L.S. Fellow of the Royal College of Physicians in Edinburgh, and of the Royal College of Surgeons in London; Member of the Royal Medical Society in Edinburgh, of the Medical and Chirurgical Society in London, of the Society of Emulation at Paris, and of the Wernerian Natural History Society; and Physician to the Plymouth Eye Dispensary.

### NOTICES TO CORRESPONDENTS.

PAPERS *preparing for the Press:*—Mr. HAWKINS *on Ulcers of the Larynx* (concluded). Dr. HARRISON *on Paraplegia from an uncommon Cause.* Mr. WADE'S *Contributions to Morbid Anatomy.* Mr. JEFFREYS' *Case of Neuralgia.* Mr. BROUGHTON *on the Use of Whiskers in the Cat Tribe.* Dr. FORBES *on Laryngitis.* Mr. JOHNSON *on Fever.* Mr. TOMS' *Case of Tetanus successfully treated with Oil of Turpentine.* Dr. GREGORY *on the Diseases prevalent in London.*

